# Prone position ventilation combined with high-flow nasal cannula oxygen therapy in patients with pulmonary infection: a retrospective study on evidence-based nursing practice

**DOI:** 10.3389/fmed.2026.1874462

**Published:** 2026-07-06

**Authors:** Wenjun Lai, Jing Fei, Ye Gao, Yan He

**Affiliations:** 1 Department of Respiratory and Critical Care Medicine, Shanghai Pulmonary Hospital Affiliated to Tongji University, Shanghai, China; 2 Department of Respiratory and Critical Care Medicine, Shanghai Fourth People’s Hospital, Shanghai, China

**Keywords:** evidence-based nursing, high-flow nasal cannula, prone position ventilation, pulmonary infection, retrospective study

## Abstract

**Background:**

Pulmonary infections are a common and serious clinical condition, particularly affecting critically ill patients, and effective respiratory management is crucial for recovery. This study examined the clinical and nursing outcomes associated with prone position ventilation (PPV) combined with high-flow nasal cannula (HFNC) oxygen therapy in patients with pulmonary infection.

**Methods:**

We conducted a retrospective analysis of 200 patients with pulmonary infection admitted to two tertiary hospitals between January 2022 and January 2024. On the basis of the respiratory support actually received during hospitalization (non-randomized allocation), patients were assigned to an observation group (*n* = 100; PPV combined with HFNC) or a control group (*n* = 100; conventional oxygen therapy); the two groups were frequency-matched for age and baseline disease severity. Outcomes included the oxygenation index (PaO2/FiO2 ratio, SpO2), respiratory rate (RR), length of hospital stay, complication and ICU-admission rates, and nurse-assessed comfort and compliance. Longitudinal oxygenation data were analysed with repeated-measures analysis of variance (ANOVA) and Bonferroni-corrected post-hoc comparisons, and prespecified multivariable-adjusted and sensitivity analyses were performed.

**Results:**

Over the first 72 h, the observation group showed greater improvements in PaO_2_/FiO_2_ ratio and SpO_2_ and a greater reduction in RR than the control group; the adjusted between-group difference in PaO_2_/FiO_2_ ratio at 72 h was 48.6 mmHg (95% CI, 41.5 to 55.7). Nurse-assessed comfort and compliance scores were higher in the observation group, and total complications (9.0% vs. 45.0%), ICU admission (5.0% vs. 19.0%) and length of stay (8.7 ± 2.4 vs. 12.0 ± 2.9 days) were lower; 30-day readmission did not differ. Results were consistent in multivariable-adjusted, propensity-score–adjusted, ICU-excluded and temporal sensitivity analyses.

**Conclusion:**

In this retrospective, non-randomized cohort, PPV combined with HFNC oxygen therapy was associated with better respiratory outcomes, fewer complications, shorter hospitalization and higher nurse-assessed comfort and compliance than conventional oxygen therapy. Because treatment allocation was not randomized, these associations cannot establish causality, and prospective randomized trials are needed to confirm the findings in larger cohorts.

## Introduction

Pulmonary infection, a prevalent cause of acute respiratory failure, presents a significant global health challenge, leading to substantial morbidity, mortality, and healthcare resource utilization (1). Patients with severe pulmonary infection often develop hypoxemic respiratory failure, characterized by impaired gas exchange and increased work of breathing, which can necessitate advanced respiratory support (2). Conventional oxygen therapy is frequently inadequate in these settings, prompting the exploration of more effective non-invasive respiratory strategies to avoid intubation and reduce complications (3). Recent global studies have highlighted the increasing burden of pulmonary infections in acute hypoxemic respiratory failure, particularly in immunocompromised populations, underscoring the need for optimized non-invasive strategies to mitigate intubation risks and improve survival (4).

High-flow nasal cannula (HFNC) oxygen therapy has emerged as a widely adopted and beneficial intervention for acute hypoxemic respiratory failure across various etiologies, including pulmonary infections (5). HFNC delivers heated and humidified oxygen at high flow rates, offering several physiological advantages over conventional oxygen therapy, such as reducing anatomical dead space, generating positive end-expiratory pressure, maintaining a constant fraction of inspired oxygen (FiO_2_), and improving mucociliary clearance (6, 7). Evidence suggests that HFNC can reduce the need for mechanical ventilation, shorten hospital stays, and improve patient comfort in selected populations (8, 9). Beyond the COVID-19 setting, systematic reviews and meta-analyses of acute hypoxemic respiratory failure of mixed and predominantly non-COVID aetiology have reported that, compared with conventional oxygen therapy, HFNC is associated with a lower risk of intubation and of escalation of respiratory support (10, 11), and in patients with bacterial or viral pulmonary infection causing acute hypoxemia HFNC has been shown to lower intubation rates and improve oxygenation relative to standard therapy (12).

Concurrently, prone position ventilation (PPV), initially recognized for its efficacy in severe acute respiratory distress syndrome among intubated patients, has gained increasing attention for its potential benefits in non-intubated, spontaneously breathing patients with hypoxemia (13). The physiological rationale for prone positioning involves improving ventilation-perfusion matching, promoting alveolar recruitment in previously collapsed dorsal lung regions, and facilitating redistribution of lung edema, thereby enhancing oxygenation and reducing atelectasis (14, 15). Recent studies indicate that awake prone positioning can improve oxygenation and potentially reduce the need for intubation in patients with acute hypoxemic respiratory failure, particularly during the COVID-19 pandemic (16, 17). Meta-analyses from 2021 onwards have reported that awake prone positioning in non-intubated patients with hypoxemic failure due to infection reduces intubation needs by improving ventilation-perfusion ratios, with sustained effects in diverse clinical settings (18).

While both HFNC and PPV have independently demonstrated advantages in managing respiratory compromise, the combined application of these two non-invasive strategies, particularly within the framework of evidence-based nursing practice for patients with pulmonary infection, remains an area requiring further investigation. The integration of these modalities may optimize respiratory outcomes by leveraging the strengths of each: HFNC provides continuous, optimized oxygen delivery and respiratory support, while prone positioning enhances lung mechanics and gas exchange (19, 20). Effective nursing care is paramount in the successful implementation of such combined therapies, ensuring patient comfort, compliance, and vigilant monitoring for potential complications (21, 22). Emerging evidence indicates that combining HFNC with prone positioning in spontaneously breathing patients with infection-related respiratory failure may augment oxygenation and decrease progression to invasive ventilation, with nursing-led protocols playing a critical role in adherence and safety (23), and that barriers to patient compliance, such as discomfort, can be mitigated through evidence-based nursing interventions (24). It should be noted, however, that much of this evidence is derived from COVID-19 cohorts; its generalizability to the broader population of non-COVID pulmonary infections remains to be confirmed, and the present study was designed to help address this gap.

Therefore, this retrospective study aimed to examine the clinical and nursing outcomes associated with PPV combined with HFNC oxygen therapy in patients with pulmonary infection in a real-world clinical setting. We hypothesized that, compared with conventional oxygen therapy, this combined approach managed through evidence-based nursing practices would be associated with better oxygenation and respiratory parameters, higher patient comfort and compliance, and lower complication rates and need for intensive care. Because the analysis is retrospective and treatment allocation was not randomized, all findings are interpreted as associations rather than treatment effects.

## Methods

### Study design and setting

This retrospective observational study was conducted in the Department of Respiratory and Critical Care Medicine at Shanghai Pulmonary Hospital (a tertiary care center specializing in respiratory diseases) and the Department of Respiratory and Critical Care Medicine at Shanghai Fourth People’s Hospital (a tertiary care center), both located in Shanghai, China, from January 2022 to January 2024. The study was approved by the institutional ethics committees of both hospitals, and the requirement for informed consent was waived owing to the retrospective design. The study is reported in accordance with the STROBE (Strengthening the Reporting of Observational Studies in Epidemiology) statement for cohort studies.

### Patient selection and group allocation

Patients diagnosed with pulmonary infection admitted to the two participating departments during the study period were screened. Inclusion criteria were: (1) age ≥18 years; (2) a diagnosis of pulmonary infection confirmed by clinical symptoms (e.g., fever, cough, dyspnea), radiographic evidence (e.g., consolidation or ground-glass opacities on chest computed tomography), and microbiological evidence (e.g., positive sputum culture or polymerase chain reaction); and (3) receipt of PPV combined with HFNC oxygen therapy or of conventional oxygen therapy as the primary non-invasive respiratory support. Exclusion criteria were: (1) severe cardiac insufficiency or hemodynamic instability; (2) contraindications to prone positioning (e.g., spinal injury, recent thoracic or abdominal surgery); and (3) incomplete core medical records.

Group allocation was not randomized. Patients were assigned to the observation group (PPV + HFNC) or the control group (conventional oxygen therapy) according to the respiratory support actually delivered during hospitalization, which was determined by the attending physician on the basis of clinical judgement and bedside resource availability rather than by a pre-specified protocol. To reduce baseline imbalance, the two groups were frequency-matched on age (in 10-year strata) and baseline disease severity, defined by the baseline PaO_2_/FiO_2_ category (100–200 vs. > 200 mmHg); during matching, patients in over-represented strata were excluded so that the two groups had comparable age and severity distributions. The numbers screened, eligible, excluded, and retained at each stage are reported in the Results (Participants and participant flow).

All patients received guideline-based pharmacological treatment of the underlying infection, including empirical antibiotics adjusted according to microbiological results and, where clinically indicated, systemic corticosteroids and standard supportive care. The time from admission to initiation of respiratory support, the proportion of patients receiving empirical antibiotics and systemic corticosteroids, and the distribution of infection syndromes (community-acquired vs. hospital-acquired pneumonia) and predominant causative pathogens (bacterial, viral, fungal, and mixed or undetermined) were recorded for both groups and are presented in Table 1.

**Table 1:** Baseline characteristics of the control and observation groups (*n* = 100 each).

Variable	Control group (*n* = 100)	Observation group (*n* = 100)	*p*-value
Demographics			
Age (years, mean ± SD)	62.3 ± 12.1	63.5 ± 11.4	0.473
Sex [male, *n* (%)]	58 (58.0)	55 (55.0)	0.669
BMI (kg/m^2^, mean ± SD)	24.5 ± 3.2	25.1 ± 3.6	0.219
Smoking history, *n* (%)	41 (41.0)	45 (45.0)	0.568
Comorbidities, *n* (%)			
Hypertension	39 (39.0)	42 (42.0)	0.663
Diabetes mellitus	27 (27.0)	30 (30.0)	0.639
Coronary artery disease	20 (20.0)	23 (23.0)	0.606
COPD	24 (24.0)	27 (27.0)	0.622
Chronic kidney disease	16 (16.0)	18 (18.0)	0.713
Clinical parameters at baseline			
PaO_2/_FiO_2_ ratio (mmHg, mean ± SD)	192.54 ± 23.3	195.46 ± 22.1	0.363
SpO_2_ (%, mean ± SD)	89.66 ± 2.7	88.92 ± 3.4	0.088
Respiratory rate (breaths/min, mean ± SD)	25.03 ± 3.8	24.66 ± 3.51	0.481
Heart rate (beats/min, mean ± SD)	93.7 ± 10.2	94.9 ± 11.1	0.426
Systolic blood pressure (mmHg, mean ± SD)	130.8 ± 15.6	132.2 ± 14.9	0.517
Diastolic blood pressure (mmHg, mean ± SD)	78.3 ± 8.4	79.5 ± 8.2	0.302
Laboratory findings at baseline			
WBC count (×10^9^/L, mean ± SD)	10.9 ± 2.8	11.2 ± 2.9	0.460
CRP (mg/L, mean ± SD)	48.6 ± 15.7	50.1 ± 16.4	0.508
PCT (ng/mL, median (IQR))	0.35 (0.15–0.92)	0.40 (0.18–0.95)	0.342
Serum creatinine (μmol/L, mean ± SD)	76.2 ± 15.4	77.8 ± 14.7	0.451
Chest imaging findings, *n* (%)			
Unilateral pneumonia	31 (31.0)	28 (28.0)	0.646
Bilateral pneumonia	69 (69.0)	72 (72.0)	0.646
Disease severity, treatment and infection profile			
Baseline PaO_2_/FiO_2_ 100–200 mmHg, *n* (%)	60 (60.0)	57 (57.0)	0.663
Baseline PaO_2_/FiO_2_ ≥ 200 mmHg, *n* (%)	40 (40.0)	43 (43.0)	0.663
Time to respiratory-support initiation (days, mean ± SD)	1.8 ± 0.9	1.7 ± 0.8	0.412
Empirical antibiotics, *n* (%)	100 (100.0)	100 (100.0)	1.000
Systemic corticosteroids, *n* (%)	38 (38.0)	41 (41.0)	0.665
Community-acquired pneumonia, *n* (%)	67 (67.0)	70 (70.0)	0.644
Hospital-acquired pneumonia, *n* (%)	33 (33.0)	30 (30.0)	0.644
Predominant pathogen – bacterial, *n* (%)	70 (70.0)	68 (68.0)	0.984
Predominant pathogen – viral, *n* (%)	21 (21.0)	22 (22.0)	0.984
Predominant pathogen – fungal, *n* (%)	5 (5.0)	6 (6.0)	0.984
Predominant pathogen – mixed/undetermined, *n* (%)	4 (4.0)	4 (4.0)	0.984

### Intervention protocol

Patients in the observation group received PPV combined with HFNC oxygen therapy. HFNC was initiated at 40–60 L/min, with flow and FiO_2_ titrated every 4–6 h to maintain SpO_2_ ≥ 92% (up to a maximum flow of 70 L/min). The prone position was maintained for at least 12 h per day, typically in 2–4 h sessions, adjusted according to patient tolerance and clinical response, with regular positional adjustments to prevent pressure injuries. The control group received conventional oxygen therapy (nasal cannula or simple face mask) with FiO_2_ adjusted to maintain SpO_2_ ≥ 92%. Nursing care in both groups was standardized according to evidence-based nursing guidelines (e.g., ATS/ESICM recommendations), including close monitoring of vital signs, respiratory pattern, and oxygenation status, and regular skin and airway management. Patients admitted to the intensive care unit (ICU) continued the respiratory strategy of their index group; any escalation to non-invasive positive-pressure ventilation or invasive mechanical ventilation was recorded as treatment failure, and these patients were analysed within their originally assigned group. No additional cross-over respiratory interventions that differed systematically between the groups were applied.

### Data collection and outcomes

Clinical data were extracted from electronic medical records by two independent researchers, with discrepancies resolved by consensus. Data included demographic information (age, sex, comorbidities), the oxygenation index (arterial partial pressure of oxygen/fraction of inspired oxygen [PaO_2_/FiO_2_ ratio] and SpO_2_), vital signs, duration of oxygen therapy, length of hospital stay, ICU admission, escalation of respiratory support, 30-day readmission, the incidence of adverse events (e.g., pressure injuries, nasal irritation, respiratory distress), and nursing records of comfort and compliance. The PaO_2_/FiO_2_ ratio, SpO_2_, and respiratory rate were recorded at baseline (before intervention) and at 24, 48, and 72 h after initiation of therapy. Analyses were based on complete cases; patients with incomplete core records were excluded at selection, and for the few isolated missing follow-up measurements within the analytic cohort (<2% of data points at any single time point) an available-case approach was used without imputation.

### Assessment of comfort and compliance

Patient comfort and treatment compliance were assessed by the bedside nursing team using a standardized institutional nursing-assessment rubric scored from 0 to 100%, with higher scores indicating greater comfort and better adherence. The comfort score incorporated patient-reported dyspnoea, tolerance of the respiratory-support interface, and ability to rest. The compliance score reflected adherence to the respiratory-support plan actually prescribed for each patient—operationalized as the proportion of the prescribed therapy time that was completed without unplanned interruption or refusal—and was therefore defined identically for both groups (prescribed PPV + HFNC time in the observation group and prescribed conventional-oxygen time in the control group). Assessments were performed once daily by trained respiratory nurses and were extracted from the electronic nursing records by two independent researchers. The rubric is an institutional tool that has not undergone formal psychometric validation, and assessors were not blinded to group allocation.

### Statistical analysis

A sample size of 200 patients was estimated to provide 80% power at a two-sided *α* of 0.05 to detect a standardized between-group difference of 0.5 in the PaO_2_/FiO_2_ ratio, based on prior studies. Continuous variables were expressed as mean ± standard deviation (SD) or median (interquartile range, IQR) according to normality (Shapiro–Wilk test) and compared with the Student *t*-test or the Mann–Whitney U test. Categorical variables were expressed as frequencies (percentages) and compared with the chi-square test, or Fisher’s exact test when expected cell counts were less than 5.

Outcomes were organized into prespecified families. The primary outcome was the PaO_2_/FiO_2_ ratio at 72 h; key secondary outcomes were SpO_2_ and respiratory rate over time, total complications, ICU admission, length of hospital stay, 30-day readmission, and nurse-assessed comfort and compliance. Longitudinal oxygenation parameters (PaO_2_/FiO_2_ ratio, SpO_2_, and respiratory rate) were analysed with repeated-measures ANOVA (group × time), with Bonferroni-corrected *post-hoc* tests for between-group comparisons at each time point. To control the family-wise type I error rate across the multiple secondary outcomes, the Bonferroni–Holm procedure was applied within the secondary-outcome family, and only comparisons that remained significant after correction were interpreted as statistically significant. Between-group differences for the primary and key continuous outcomes are reported with 95% confidence intervals (CIs). Exact *p* values are reported throughout, and values below 0.001 are reported as *p* < 0.001.

To assess the influence of residual confounding, two prespecified adjusted analyses were performed: (i) multivariable linear regression for the 72-h PaO_2_/FiO_2_ ratio and multivariable logistic regression for total complications and ICU admission, adjusting for age, sex, body mass index, smoking, hypertension, diabetes mellitus, chronic obstructive pulmonary disease, baseline PaO_2_/FiO_2_ ratio, infection classification, and systemic-corticosteroid use; and (ii) a propensity-score–adjusted analysis using the same covariates. Three prespecified sensitivity analyses were performed: exclusion of patients admitted to the ICU, restriction to the calendar year with the highest enrolment, and adjustment for calendar year of enrolment to address potential temporal confounding. Analyses were performed with SPSS v26.0 (IBM Corp., Armonk, NY, USA), and figures were prepared with GraphPad Prism v9.0 (GraphPad Software Inc., San Diego, CA, USA). A two-sided *p* < 0.05 (after correction, where applicable) was considered statistically significant.

## Results

### Participants and participant flow

Of 284 patients screened, 47 were excluded before matching (18 for haemodynamic instability or severe cardiac insufficiency, 14 for contraindications to prone positioning, and 15 for incomplete core records), leaving 237 eligible patients (118 who had received PPV + HFNC and 119 who had received conventional oxygen therapy). Frequency matching on age and baseline severity excluded 37 patients in over-represented strata (18 from the PPV + HFNC group and 19 from the conventional-therapy group, distributed across age and severity strata), yielding the analytic cohort of 200 patients (100 per group).

### Baseline characteristics

Baseline characteristics of the control group (*n* = 100) and the observation group (*n* = 100) are summarized in Table 1. No statistically significant differences were observed between the two groups (all *p* > 0.05).

Demographic features were similar, including age (62.3 ± 12.1 vs. 63.5 ± 11.4 years, *p* = 0.473), sex (58.0% vs. 55.0% male, *p* = 0.669), body mass index (24.5 ± 3.2 vs. 25.1 ± 3.6 kg/m^2^, *p* = 0.219), and smoking history (41.0% vs. 45.0%, *p* = 0.568). Comorbidities including hypertension (39.0% vs. 42.0%, *p* = 0.663), diabetes mellitus (27.0% vs. 30.0%, *p* = 0.639), coronary artery disease (20.0% vs. 23.0%, *p* = 0.606), chronic obstructive pulmonary disease (COPD; 24.0% vs. 27.0%, *p* = 0.622), and chronic kidney disease (16.0% vs. 18.0%, *p* = 0.713) did not differ.

Baseline clinical parameters were comparable, including the PaO_2_/FiO_2_ ratio (192.54 ± 23.3 vs. 195.46 ± 22.1 mmHg, *p* = 0.363), SpO_2_ (89.66 ± 2.7 vs. 88.92 ± 3.4%, *p* = 0.088), respiratory rate (25.03 ± 3.8 vs. 24.66 ± 3.51 breaths/min, *p* = 0.481), heart rate (93.7 ± 10.2 vs. 94.9 ± 11.1 beats/min, *p* = 0.426), systolic blood pressure (130.8 ± 15.6 vs. 132.2 ± 14.9 mmHg, *p* = 0.517), and diastolic blood pressure (78.3 ± 8.4 vs. 79.5 ± 8.2 mmHg, *p* = 0.302). Laboratory findings, including white blood cell count (10.9 ± 2.8 vs. 11.2 ± 2.9 × 10^9^/L, *p* = 0.460), C-reactive protein (48.6 ± 15.7 vs. 50.1 ± 16.4 mg/L, *p* = 0.508), procalcitonin (median 0.35 [IQR 0.15–0.92] vs. 0.40 [0.18–0.95] ng/mL, *p* = 0.342), and serum creatinine (76.2 ± 15.4 vs. 77.8 ± 14.7 μmol/L, *p* = 0.451), were similar, as were chest-imaging findings (unilateral pneumonia 31.0% vs. 28.0% and bilateral pneumonia 69.0% vs. 72.0%, *p* = 0.646).

Baseline disease severity (PaO_2_/FiO_2_ 100–200 vs. > 200 mmHg, *p* = 0.663), time from admission to initiation of respiratory support (1.8 ± 0.9 vs. 1.7 ± 0.8 days, *p* = 0.412), the proportion receiving empirical antibiotics (100.0% vs. 100.0%, *p* = 1.000) and systemic corticosteroids (38.0% vs. 41.0%, *p* = 0.665), the distribution of infection syndromes (community-acquired pneumonia 67.0% vs. 70.0%; hospital-acquired pneumonia 33.0% vs. 30.0%, *p* = 0.644), and the predominant causative pathogens (bacterial 70.0% vs. 68.0%; viral 21.0% vs. 22.0%; fungal 5.0% vs. 6.0%; mixed or undetermined 4.0% vs. 4.0%, *p* = 0.984) did not differ significantly between groups (Table 1).

### Improvement in oxygenation and respiratory rate

**Figure 1:**
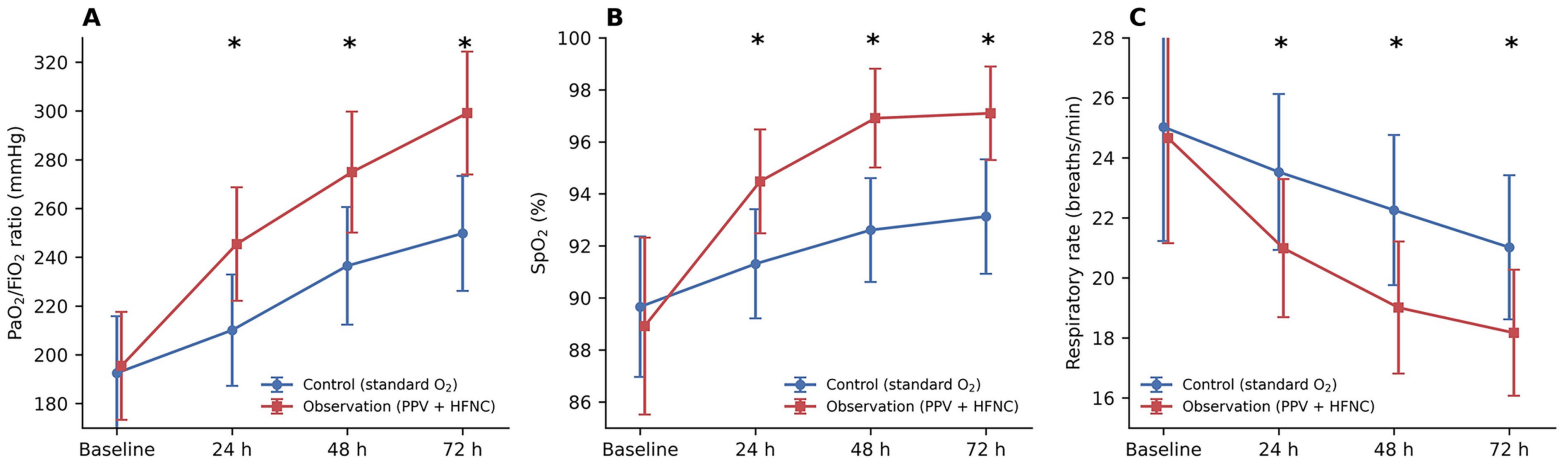
Oxygenation and respiratory rate over time. **(A)** PaO2/FiO2 ratio (mmHg), **(B)** SpO_2_ (%), and **(C)** respiratory rate (breaths/min) in the control group (conventional oxygen therapy) and the observation group (PPV + HFNC) at baseline and at 24, 48, and 72 h. Data are mean ± SD (*n* = 100 per group). *p < 0.001 versus control at the same time point (Bonferroni-corrected *post-hoc* comparison within the repeated-measures ANOVA model). Baseline values did not differ significantly between groups (*p* > 0.05).

Changes in oxygenation parameters and respiratory rate over 72 h are shown in Figure 1. The mean PaO_2_/FiO_2_ ratio (Figure 1A) in the control group rose from 192.54 ± 23.25 mmHg at baseline to 210.11 ± 22.8 mmHg at 24 h, 236.51 ± 24.1 mmHg at 48 h, and 249.79 ± 23.6 mmHg at 72 h, whereas in the observation group it rose from 195.46 ± 22.1 mmHg at baseline to 245.42 ± 23.3 mmHg at 24 h, 274.95 ± 24.8 mmHg at 48 h, and 299.12 ± 25.2 mmHg at 72 h. The between-group difference at 72 h was 49.33 mmHg (95% CI, 42.56 to 56.10; *p* < 0.001).

SpO_2_ (Figure 1B) in the control group rose from 89.66% ± 2.7% at baseline to 91.31% ± 2.1%, 92.61% ± 2.0%, and 93.13% ± 2.2% at 24, 48, and 72 h, respectively, and in the observation group from 88.92% ± 3.4% to 94.48% ± 2.0%, 96.91% ± 1.9%, and 97.10% ± 1.8% at the same time points; the between-group difference at 72 h was 3.97% (95% CI, 3.41 to 4.53; *p* < 0.001).

Respiratory rate (Figure 1C) in the control group decreased from 25.03 ± 3.8 breaths/min at baseline to 23.53 ± 2.6, 22.26 ± 2.5, and 21.02 ± 2.4 breaths/min at 24, 48, and 72 h, and in the observation group from 24.66 ± 3.5 to 20.99 ± 2.3, 19.01 ± 2.2, and 18.17 ± 2.1 breaths/min; the between-group difference at 72 h was −2.85 breaths/min (95% CI, −3.48 to −2.22; *p* < 0.001). All between-group comparisons at 24, 48, and 72 h remained statistically significant after Bonferroni correction (*p* < 0.001).

### Comfort and compliance

**Figure 2:**
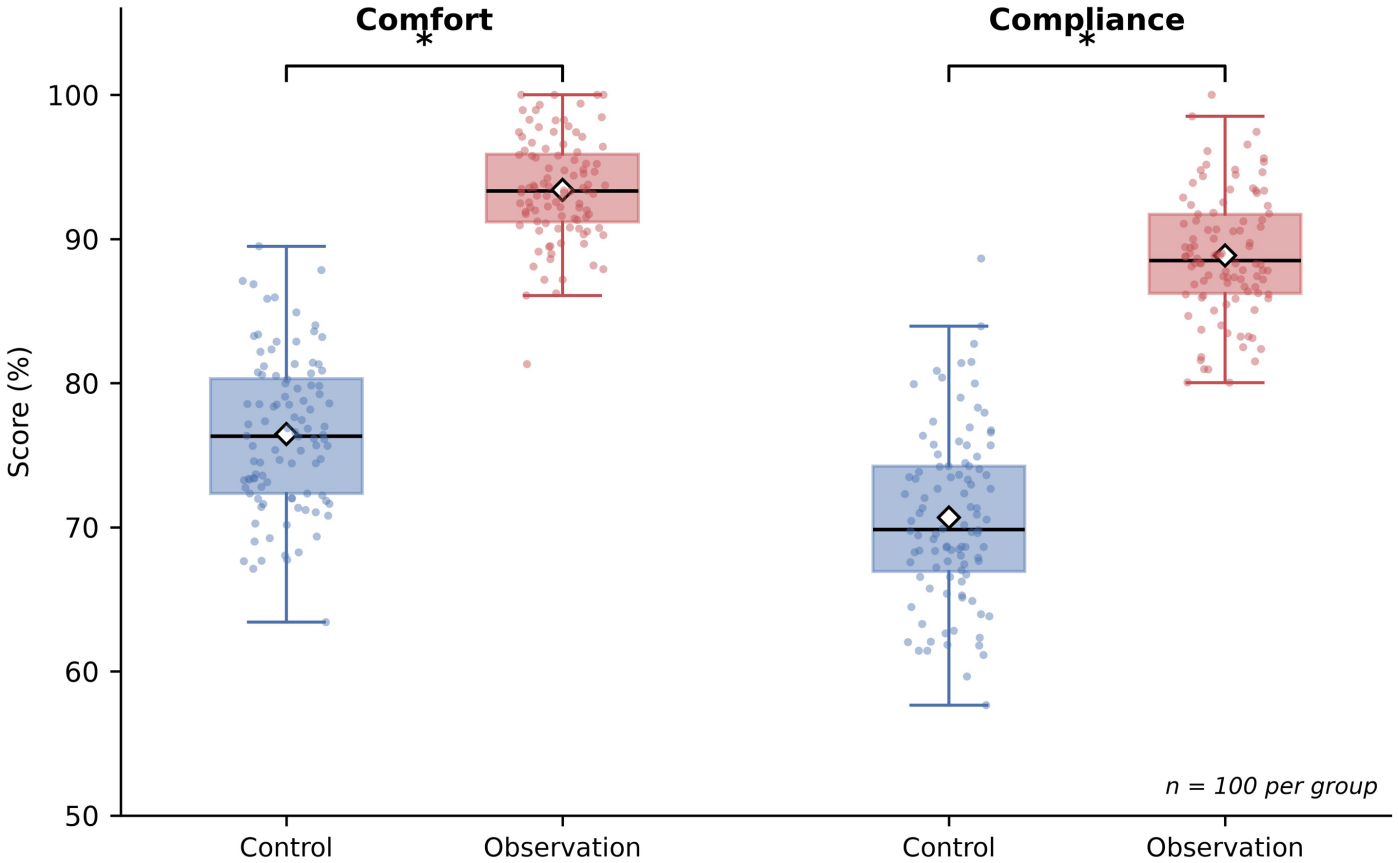
Nurse-assessed comfort and compliance scores. Box plots of comfort and compliance scores (0%–100%) in the control group (conventional oxygen therapy) and the observation group (PPV + HFNC) (*n* = 100 per group). Boxes show the median and interquartile range, whiskers extend to 1.5 times the interquartile range, the white diamond marks the mean, and individual data points are overlaid. The observation group had higher comfort (mean 93.0 ± 4.0% vs. 76.0 ± 6.0%) and compliance (89.0 ± 4.0% vs. 70.0 ± 6.0%) scores. *p < 0.001 between groups (Student *t*-test).

Nurse-assessed comfort and compliance scores are shown in Figure 2. The observation group had a higher comfort score than the control group (93.0% ± 4.0%; median 93 [IQR 90–96] vs. 76.0% ± 6.0%; median 76 [72–80]; between-group difference 17.0, 95% CI 15.59 to 18.41; *p* < 0.001) and a higher compliance score (89.0 ± 4.0%; median 89 [86–92] vs. 70.0 ± 6.0%; median 70 [66–74]; between-group difference 19.0, 95% CI 17.59 to 20.41; *p* < 0.001). Both comparisons remained significant after correction for multiple comparisons.

### Complications and hospital stay

Complications and hospitalization outcomes are summarized in Table 2. Individual complications were less frequent in the observation group than in the control group: pressure ulcers (2.0% vs. 11.0%, *p* = 0.018), severe pneumonia progression (1.0% vs. 13.0%, *p* = 0.001), and nosocomial infection (1.0% vs. 15.0%, *p* < 0.001); device intolerance or therapy withdrawal (2.0% vs. 5.0%, *p* = 0.445) and aspiration or airway obstruction (3.0% vs. 10.0%, *p* = 0.082) were numerically lower but not statistically significant. The total complication rate, defined as the proportion of patients with at least one complication, was 9.0% in the observation group versus 45.0% in the control group (*p* < 0.001). Because some patients had more than one event, the sum of the individual complication counts in the control group (11 + 13 + 15 + 5 + 10 = 54 events) exceeds the number of affected patients (45): nine control-group patients each had two or more concurrent complications, whereas in the observation group the nine events occurred in nine separate patients with no overlap.

**Table 2:** Complication rates and hospital stay in the control and observation groups (*n* = 100 each).

Variable	Control group (*n* = 100)	Observation group (*n* = 100)	*p*-value
Complications, *n* (%)			
Pressure ulcers	11 (11.0)	2 (2.0)	0.018
Severe pneumonia progression	13 (13.0)	1 (1.0)	0.001
Nosocomial infections	15 (15.0)	1 (1.0)	<0.001
Device intolerance / therapy withdrawal	5 (5.0)	2 (2.0)	0.445
Aspiration or airway obstruction	10 (10.0)	3 (3.0)	0.082
Total complications (≥1 per patient)	45 (45.0)	9 (9.0)	<0.001
Hospitalization outcomes			
Hospital stay (days), mean ± SD	12.0 ± 2.9	8.7 ± 2.4	<0.001
ICU admission, *n* (%)	19 (19.0)	5 (5.0)	0.004
Treatment failure (escalation beyond index strategy), *n* (%)	17 (17.0)	4 (4.0)	0.003
30-day readmission, *n* (%)	13 (13.0)	11 (11.0)	0.828

The mean length of hospital stay was shorter in the observation group than in the control group (8.7 ± 2.4 vs. 12.0 ± 2.9 days; mean difference −3.3 days, 95% CI − 4.04 to −2.56; *p* < 0.001), and ICU admission was less frequent (5.0% vs. 19.0%, *p* = 0.004). The 30-day readmission rate did not differ significantly between the two groups (11.0% vs. 13.0%, *p* = 0.828).

### ICU management and treatment failure

Among the 19 control-group and 5 observation-group patients admitted to the ICU, escalation beyond the index respiratory strategy (treatment failure) occurred in 17 control-group patients (11 escalated to non-invasive positive-pressure ventilation and 6 to invasive mechanical ventilation) and in 4 observation-group patients (3 and 1, respectively); the remaining ICU patients (2 control, 1 observation) continued their index strategy. The treatment-failure rate was 17.0% in the control group versus 4.0% in the observation group (*p* = 0.003) (Table 2).

### Adjusted and sensitivity analyses

In multivariable analysis, the adjusted between-group difference in the 72-h PaO_2_/FiO_2_ ratio was 48.6 mmHg (95% CI, 41.5 to 55.7; *p* < 0.001), and the adjusted odds ratios for the observation versus control group were 0.12 (95% CI, 0.05 to 0.27; *p* < 0.001) for total complications and 0.22 (95% CI, 0.08 to 0.62; *p* = 0.004) for ICU admission; propensity-score–adjusted estimates were concordant (72-h PaO_2_/FiO_2_ difference 48.1 mmHg, 95% CI 40.8 to 55.4). Excluding the patients admitted to the ICU (control *n* = 81, observation *n* = 95), the 72-h between-group difference in the PaO_2_/FiO_2_ ratio remained 48.8 mmHg (95% CI, 41.9 to 55.7; *p* < 0.001), and the differences in complications and length of stay were materially unchanged. Enrolment was distributed across the study period (control vs. observation: 34 vs. 32 in 2022, 54 vs. 56 in 2023, and 12 vs. 12 in January 2024; *p* = 0.94 for the between-group distribution), and restricting the analysis to 2023 or additionally adjusting for calendar year of enrolment did not materially change any between-group estimate.

## Discussion

In this retrospective, non-randomized cohort of patients with pulmonary infection, PPV combined with HFNC oxygen therapy was associated with more favourable clinical outcomes than conventional oxygen therapy, including greater improvements in the PaO2/FiO2 ratio and SpO_2_, a greater reduction in respiratory rate, higher nurse-assessed comfort and compliance, fewer complications, shorter hospital stay, and lower ICU admission, with no significant difference in 30-day readmission. These associations were consistent across multivariable-adjusted, propensity-score–adjusted, ICU-excluded, and temporal sensitivity analyses, which strengthens their internal consistency, although they cannot establish causality.

Our observations are consistent with emerging evidence on the combined use of PPV and HFNC in acute hypoxemic respiratory failure of infectious etiology (25). The greater improvement in PaO_2_/FiO_2_ ratio and SpO_2_ over 72 h is physiologically plausible: PPV promotes alveolar recruitment in dorsal lung regions, reducing ventilation-perfusion mismatch and atelectasis, while HFNC generates positive end-expiratory pressure, washes out anatomical dead space, and delivers a stable FiO_2_. The more rapid decline in respiratory rate is compatible with a reduced work of breathing, in line with studies of awake prone positioning combined with HFNC in severe pneumonia or ARDS (26). Because much of the supporting literature derives from COVID-19 cohorts, the consistency of our findings in a broader, predominantly non-COVID pulmonary-infection population—together with non-COVID meta-analytic evidence that HFNC reduces intubation and escalation relative to conventional oxygen (10, 11)—adds incremental, though still non-randomized, support for these strategies.

The higher comfort and compliance scores in the observation group may reflect the non-invasive nature of HFNC, which minimizes nasal irritation, and the ability of prone positioning to redistribute pressure and enhance secretion drainage, thereby reducing dyspnoea and discomfort (27). Adequate adherence is important because poor compliance can limit the duration of effective therapy, and our findings are consistent with reports that nursing-led protocols for awake prone positioning mitigate barriers such as discomfort (28). These patient-reported outcomes should nonetheless be interpreted cautiously: the rubric used has not been formally validated and the assessors were not blinded, so information bias cannot be excluded.

The lower complication rates in the observation group, including fewer pressure ulcers, less severe pneumonia progression, and fewer nosocomial infections, are compatible with a favourable safety profile. Because the patients were not intubated, the lower risk of ventilator-associated complications such as barotrauma is most plausibly attributable to the avoidance of intubation and invasive mechanical ventilation rather than to a direct effect of PPV + HFNC on the lung parenchyma, consistent with reports that early prone positioning with HFNC may prevent progression to invasive support (29). Shorter hospital stay and fewer ICU admissions may be associated with more efficient resource use, although this hypothesis would require formal health-economic evaluation. The absence of a difference in 30-day readmission suggests that any benefit is concentrated in the acute phase and that longer-term outcomes warrant dedicated follow-up.

Clinically, these findings support the further evaluation of PPV combined with HFNC within evidence-based nursing protocols for patients with pulmonary infection and hypoxaemia, particularly in non-intubated settings, where the combination might serve as a bridge that reduces escalation to invasive ventilation (30); they are not, however, sufficient to establish superiority over conventional therapy.

Several limitations should be acknowledged. First, the study was retrospective and treatment allocation was not randomized, so selection bias and confounding by indication cannot be excluded despite baseline comparability and frequency matching; in particular, clinicians may have preferentially offered PPV + HFNC to more cooperative or less severely ill patients. Second, although multivariable and propensity-score–adjusted analyses were concordant with the unadjusted results, these methods can address only measured covariates, and residual unmeasured confounding may persist; relatedly, the magnitude of some between-group differences is larger than is typically observed in retrospective cohorts, and although it persisted after multivariable and propensity-score adjustment it may partly reflect non-randomized treatment selection and unmeasured between-group differences and therefore requires confirmation in randomized controlled trials. Third, comfort and compliance were assessed with a non-validated institutional rubric by unblinded assessors, which may have introduced information bias. Fourth, the cohort was clinically heterogeneous with respect to infection etiology; although the distribution of syndromes and pathogens was comparable between groups, the study was not powered for stratified analyses by pathogen. Fifth, the study period (January 2022 to January 2024) spanned the post-COVID-19 transition, during which institutional protocols and familiarity with prone positioning may have evolved; although enrolment was distributed across the period and temporal sensitivity analyses were concordant, unmeasured temporal effects cannot be fully excluded. Sixth, both centres are tertiary hospitals in a single city, which limits generalizability, and outcomes beyond the index admission and 30-day readmission were not evaluated, precluding assessment of longer-term benefit. Finally, the retrospective, non-randomized design does not permit causal inference, and all findings should be interpreted as associations. Prospective, adequately powered randomized controlled trials in larger, multicentre cohorts are warranted to confirm these results and to explore subgroups defined by infection severity and etiology (31, 32).

## Conclusion

In this retrospective, non-randomized study, PPV combined with HFNC oxygen therapy was associated with more favourable outcomes than conventional oxygen therapy in patients with pulmonary infection, including better oxygenation and respiratory rate, higher nurse-assessed comfort and compliance, fewer complications, shorter hospital stay, and lower ICU admission, without a difference in 30-day readmission; these associations were robust to multivariable adjustment and sensitivity analyses. Because the design precludes causal inference, the combination should be regarded as a promising, patient-centred non-invasive strategy that merits confirmation in prospective randomized controlled trials before routine adoption within evidence-based nursing protocols.

## Data Availability

The original contributions presented in the study are included in the article/Supplementary material, further inquiries can be directed to the corresponding authors.
