# Supplementary material for: Prone position ventilation combined with high-flow nasal cannula oxygen therapy in patients with pulmonary infection: a retrospective study on evidence-based nursing practice
Source: Front Med (Lausanne). 2026 Jul 6;13:1874462. doi: 10.3389/fmed.2026.1874462 (PMC13381630; doi:10.3389/fmed.2026.1874462)
Supplement: Supplementary file 1 [file Table_1.docx]

**STROBE Statement — Checklist for cohort studies**

Manuscript ID 1874462

*Prone position ventilation combined with high-flow nasal cannula oxygen therapy in patients with pulmonary infection: A retrospective study on evidence-based nursing practice*

| **Item** | **Recommendation** | **Location in manuscript** |
| --- | --- | --- |
| Title & abstract — 1 | (a) design in title/abstract; (b) balanced summary | Title and Abstract; design stated as “retrospective” and “non-randomized”. |
| Background/rationale — 2 | Scientific background and rationale | Introduction, paragraphs 1–4. |
| Objectives — 3 | Specific objectives / hypotheses | Introduction, final paragraph (associative aim and hypotheses). |
| Study design — 4 | Key elements of design | Methods – Study design and setting. |
| Setting — 5 | Setting, locations, dates | Methods – Study design and setting (two tertiary hospitals, Jan 2022–Jan 2024). |
| Participants — 6 | Eligibility, sources, selection, matching | Methods – Patient selection and group allocation; Results – Participants and participant flow. |
| Variables — 7 | Outcomes, exposures, confounders | Methods – Data collection and outcomes; Statistical analysis (covariates). |
| Data sources/measurement — 8 | Sources and measurement methods | Methods – Data collection and outcomes; Assessment of comfort and compliance. |
| Bias — 9 | Efforts to address bias | Methods – frequency matching; adjusted and sensitivity analyses (Statistical analysis). |
| Study size — 10 | How study size was determined | Methods – Statistical analysis (power calculation). |
| Quantitative variables — 11 | Handling of quantitative variables | Methods – Statistical analysis (normality; mean±SD or median[IQR]). |
| Statistical methods — 12 | Methods, multiplicity, subgroups, sensitivity, missing data | Methods – Statistical analysis (repeated-measures ANOVA; Bonferroni/Bonferroni–Holm; multivariable and propensity-score–adjusted analyses; ICU-excluded and temporal sensitivity analyses; complete-/available-case handling of missing data). |
| Participants — 13 | Numbers at each stage; matching | Results – Participants and participant flow (284→237→200). |
| Descriptive data — 14 | Characteristics; missing data | Results – Baseline characteristics; Table 1. |
| Outcome data — 15 | Numbers of outcome events | Results – oxygenation, comfort/compliance, complications; Table 2; ICU treatment failure. |
| Main results — 16 | Estimates, confidence intervals, adjustment | Results – 72-h between-group differences with 95% CIs; Adjusted and sensitivity analyses. |
| Other analyses — 17 | Subgroups, interactions, sensitivity | Results – Adjusted and sensitivity analyses (multivariable, propensity-score, ICU-excluded, temporal). |
| Key results — 18 | Summary with reference to objectives | Discussion, paragraph 1. |
| Limitations — 19 | Limitations, direction and magnitude of bias | Discussion – enumerated Limitations. |
| Interpretation — 20 | Cautious overall interpretation | Discussion (paragraphs 4–5) and Conclusion. |
| Generalisability — 21 | External validity | Discussion – Limitations (two tertiary hospitals in one city). |
| Funding — 22 | Funding source / role of funders | Funding. |
